# Dysentery Treated by Nux Vomica

**Published:** 1868-08

**Authors:** William M. Cornell


### Dysentery Treated by Nux Vomica.

BY WILLIAM M. CORNELL, M. D., LL. D

Dr. Miner:—I have not forgotten my promise to write out for your valuable Journal those cases of disease of the throat and lungs, treated by inhalation of atomized fluids, which I promised you, but circumstances have hitherto prevented. You have noticed in the medical journals lately, the *treatment of dysentery by nux vomica*, as though it were something new. But, in this respect, "the thing that is, is that which has been."

In 1848, I attended, and reported the following cases, which may be found in the fourth volume, p. 295, of the *Charleston (S. C.) Medical Journal*, published in 1849. It may be useful to re-publish them in your Journal. I may add, I have treated dysentery in the same way ever since, now twenty years.

*Ten Cases of Epidemic Dysentery successfully treated by Nux Vomica.*—In August, September, and the early part of October, 1848, the dysentery prevailed more in the vicinity of Boston, and proved more fatal than it had been for many years. In the village of S——, where I was spending my nights, at this season, it was very prevalent, and, in many cases, proved fatal. The usual remedies seemed to produce no good effect. The disease run a rapid course into a putrid stage, and death ensued. This was, more especially, the case with children, among whom it prevailed more than among adults. Almost all who were attacked between the ages of six months and four years, died.

The town of S——, though elevated several hundred feet above the level of the sea, lies, nevertheless, in a valley, and the reflection of the sun from the surrounding hills produced a high temperature during the day, and the evaporation from stagnant water in the valley lowered the temperature much at night. There was often a difference of twenty degrees between the day and the night. This, in my opinion, accounted for the prevalence of the disease. I think such locations are much more liable to dysentery than those where the thermometer shows but a little difference between the day and the night.

The following was the general course of the symptoms—there were, however, some sporadic variations: At the first, slight chills, soon followed by a low grade of fever; a hard, frequent jerking, though not full pulse; frequent discharges of blood and mucus, with griping pains in the bowels, and constant pain in the loins; no pain in the head, skin rough and dry; urine high colored and scanty. The colon and rectum seemed to be the chief seat of the disease. There was little or no bile mingled with the stools. With the children, especially, the disease ran speedily into a putrid type. All the medicines usually employed in dysentery—castor oil and laudanum, opium and ipecac, acetate of lead, kino, sulphate of magnesia, sulphate of soda, tartrate of potassa and soda, comp. powder of jalap, and the whole range of diaphoretics, wild strawberry, blackberry, etc., all were tried, but apparently did no good. The physician of the village said, he did not wish to be called to a case, for they all died.

In this state of things, I was led to look around for some other medicine, and turning over all the books of my medical library—not a very small one—I hit upon the following passage in the first volume of John Samstrong's works, of London, page 419: "A friend of mine, Mr. George Vaut, of Ipswich, has tried a remedy in dysentery for sixteen years, in about two hundred cases, and the result has been successful, and so remarkably uniform, that I feel it my duty to mention the treatment here. (This was to his medical class.) This gentleman gives in dysentery, or inflammation of the mucous membrane about the colon, seven grains of nux vomica thrice, daily. It neither purges nor constipates, but removes the inflammation, and healthy evacuations follow. Mr. Vaut, who resides in London, bears similar testimony to the value of this remedy, and I strongly recommend it to your notice. I shall certainly try it in the next case I meet with. It seems to operate as a sort of specific. It was first mentioned by Hagstrem, and has been very much neglected since his day."

Upon reading the above, I immediately determined, under the circumstances above stated, to make trial of the nux. I did so, and gave it in the full dose of seven grains thrice, daily, to adults, and from one to four grains to children, in proportion to their ages. The result was most happy. Not a patient who was treated with this medicine died. It was prescribed in ten cases, within three or four weeks, and all recovered. No cathartic medicine was given, except teaspoonful doses of *bitartrate of potassa* in a few cases.

It would be presumption to say that this medicine is a perfect *specific* for dysentery in all cases. Indeed, I am far from having much confidence in *specifics* generally; but, I am constrained to say, that the above named medicine altogether exceeded my expectations, and I earnestly recommend a trial of it in dysentery.

I had one case of which I almost despaired before using the nux. But the patient recovered under its use. I hope the profession will give this medicine fair trial. I tried the *strychnine*, but it did not succeed so well as the nux.

